# Ferroelectric Resistance Switching in Epitaxial BiFeO_3_/La_0.7_Sr_0.3_MnO_3_ Heterostructures

**DOI:** 10.3390/ma16227198

**Published:** 2023-11-17

**Authors:** Hongyan Qi, Weixin Wu, Xinqi Chen

**Affiliations:** Expert Workstation for Terahertz Technology and Advanced Energy Materials and Devices, School of Physics and Mechanical & Electronical Engineering, Hubei University of Education, Wuhan 430205, China; kalxtist@gmail.com

**Keywords:** resistance switching, interface, ferroelectric polarization, Schottky barrier

## Abstract

BiFeO_3_/La_0.7_Sr_0.3_MnO_3_ (BFO/LSMO) epitaxial heterostructures were successfully synthesized by pulsed laser deposition on (001)-oriented SrTiO_3_ single-crystal substrates with Au top electrodes. Stable bipolar resistive switching characteristics regulated by ferroelectric polarization reversal was observed in the Au/BFO/LSMO heterostructures. The conduction mechanism was revealed to follow the Schottky emission model, and the Schottky barriers in high-resistance and low-resistance states were estimated based on temperature-dependent current–voltage curves. Further, the observed memristive behavior was interpreted via the modulation effect on the depletion region width and the Schottky barrier height caused by ferroelectric polarization reversal, combining with the oxygen vacancies migration near the BFO/LSMO interface.

## 1. Introduction

In recent years, nonvolatile resistive switching (RS) behavior has attracted significant attention due to its high speed, low power dissipation, and its potential applications in next-generation, ultra-high-density resistance switching random-access memories of simple devices. Conventional binary metal oxides such as TiO_2_ [1], NiO [2], ZnO [3], Cu_x_O [4], etc., have been frequently used as RS layers in memristors. However, in order to develop high-performance memory devices, ferroelectric thin films are emerging as alternatives to RS layers. Polarization switching characteristics provide new degrees of freedom to manipulate the RS behavior. They also provide a rectification current–voltage behavior, and thus reduce sneak currents in crossbar resistive random-access memory devices [5]. Ferroelectric thin films, including BTO [6], 30% Sr-doped BaTiO_3_ [7], 5% Al_0.5_Nb_0.5_O_3_-doped Pb(Zr_0.52_Ti_0.48_)O_3_ (PZT) [8], BiFeO_3_ (BFO) [9,10,11,12], Ca-doped BiFeO_3_ [13], and Hf_0.93_Y_0.07_O_2_ [14], have been investigated as the RS layer. With emerging manipulation methods, various interpretations have been proposed to understand the ferroelectric resistance switching behavior, such as the conductive filaments model [15], trapping/detrapping of the free charges and the migration of the oxygen vacancies [7], and polarization-modulated Schottky barriers [16], whereas a clear understanding of the switching mechanisms in ferroelectric memristors still poses a challenge.

As the famous room-temperature multiferroic, BFO has attracted huge interest and extensive investigation in the past decades. BFO has a high spontaneous polarization of ~100 μC/cm^2^ along the pseudocubic [111] direction with a Curie temperature of 1100 K [17,18,19]. The large spontaneous polarization and high Curie temperature make it suitable for Schottky barrier manipulation via ferroelectric polarization. La_0.7_Sr_0.3_MnO_3_ (LSMO) attracts huge research interest because it is by far the only simple perovskite manganite with a Curie temperature higher than room temperature (T_C_~370 K) [20], which exhibits promising potential application as a magnetic storage media and magnetic sensor. LSMO exhibits colossal magnetoresistance, and it is induced by the concomitance of a metal-insulator and a ferromagnetic-to-paramagnetic phase transition in a perovskite atomic structure [20,21]. The metallic nature of LSMO makes it a candidate for oxide electrodes. Both LSMO and BFO are perovskite oxides and are structurally compatible with each other with small misfit strain [22], which is helpful for the formation of the coherent heterostructure. In the BFO/LSMO bilayer, a diodelike RS behavior was observed and the retention properties were discussed comprehensively by considering the depolarization field-driven polarization relaxation [23]. This would be helpful for further understanding the conductive mechanism for the system.

In this work, we prepared an epitaxial BFO/LSMO heterostructure as the RS layer and studied the RS behavior by measuring the temperature-dependent current–voltage properties and calculating the conduction barriers. A model of ferroelectric polarization reversal modulation combining the migratory effect of the oxygen vacancies near the BFO/LSMO interface has been proposed to interpret the RS mechanism.

## 2. Experimental Section

BFO films of 150 nm thick were epitaxially grown on (001) SrTiO_3_ single-crystal substrates buffered with LSMO thin films as the bottom electrode. Both BFO and LSMO were prepared by a pulsed laser deposition technique with a 248 nm KrF excimer laser. BFO and LSMO were grown at 690 °C and 750 °C under oxygen pressures of 15 and 16 Pa, respectively. Further in situ post-annealing under a 3000 Pa oxygen partial pressure was conducted for 30 min. The phase structure was studied by X-ray diffraction (XRD; Bruker D8 Discover, Karlsruhe, Germany) using CuKα_1_ radiation (λ = 1.5406 Å). The ferroelectric polarization–electric field hysteresis loops were measured on a ferroelectric tester (Precision II, Radiant Technologies, Albuquerque, MN, USA). The local piezoresponse was measured by piezoelectric force microscopy (PFM, MFP-3D, Oxford, MS, USA). Current–voltage curves were recorded on a Keithley 2450 source meter (Tektronix Inc., Beaverton, OR, USA) using Au discs as the top electrodes.

## 3. Results and Discussion

**Figure 1:**
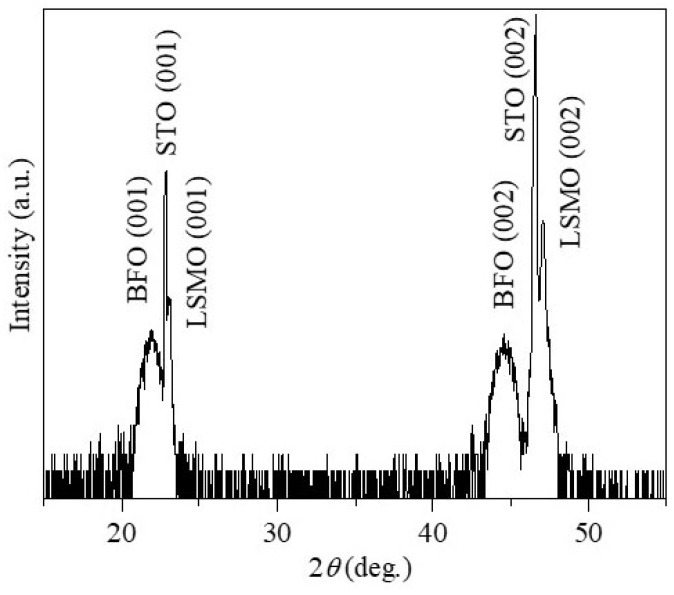
XRD pattern of the BFO/LSMO heterostructure.

Figure 1 shows a typical XRD *θ*-2*θ* pattern for the as-prepared BFO/LSMO heterostructures on the (001) STO substrate. Only (00l)-type reflections are observed, indicating predominant (001) growth along the orientation of the substrate.

**Figure 2:**
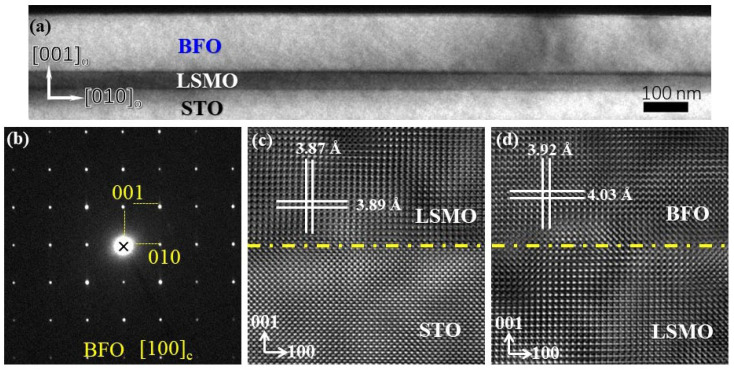
TEM image (**a**), SAED (**b**), HRTEM images near LSMO/STO (**c**), and BFO/LSMO (**d**) interface of the BFO/LSMO heterostructure.

Figure 2a displays the typical morphology of a cross-section of a BFO/LSMO heterostructure. The thickness of the BFO layer was measured to be approximately 150 nm. The relatively thicker BFO films will result in a smaller depolarization field, which may cause a fast polarization relaxation and reduce the retention property [23]. One can observe a sharp, smooth interface between the BFO/LSMO film and the STO substrate. Figure 2b shows selected area electron diffraction (SAED) patterns along the [100] c zone axis, taken from the BFO layer. The reflection patterns were indexed based on pseudocubic unit cells of BFO. The interface structures of LSMO/STO and BFO/LSMO were illustrated by the high-resolution TEM images, as shown in Figure 2c,d. One can see that the interface is coherent, without any misfit dislocations near the interface, indicating the high epitaxy and crystallinity of the BFO and LSMO films. The spacings of the lattice fringes from two directions were measured as 0.392 nm and 0.403 nm in Figure 2c, corresponding to the (001) and (010) planes of pseudocubic BiFeO_3_, respectively. Combining the diffraction and high resolution results, the epitaxial relationships between the film and the substrate can be determined to be (001) BFO//(001) LSMO//(001) STO and [010] BFO//[010] LSMO//[010] STO, which are consistent with the XRD results. A sharp and smooth interface manifests the high quality of the heterostructures and ensures that there are minimal defects, dislocations, or roughness that could disrupt the integrity of the heterostructure. The high quality of the interface structure is essential for achieving the desired resistive switching characteristics in the epitaxial heterostructure.

**Figure 3:**
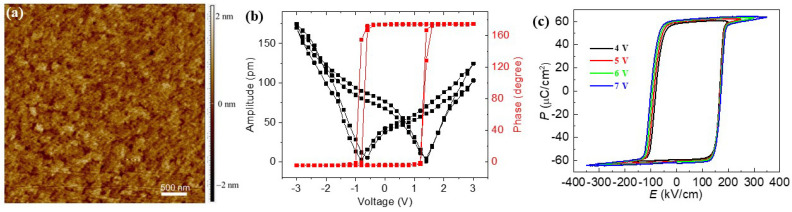
AFM image (**a**) of the BFO surface, PFM phase−voltage hysteresis and amplitude−voltage loops (**b**), and P−E hysteresis loops (**c**) of BFO films.

Figure 3a presents an AFM topography image of the BFO film on a 4 μm × 4 μm scale. The BFO film is smooth, and the root-mean-square roughness was measured to be 1.2 nm. The local piezoelectric response of the BFO thin film were studied by piezoelectric force microscopy, and the piezoresponse phase–voltage hysteresis loops and the butterfly-shaped amplitude–voltage curves are shown in Figure 3b. The apparent 180° phase flip in the phase–voltage loops confirmed the domain switching of the BFO film, while dips in the amplitude–voltage curves at −0.8 V and 1.4 V correspond to the coercive voltages of BFO films in the heterostructure. The asymmetric coercive voltages may result from the built-in fields [24,25] or the contact barriers between the ferroelectric films and the top as well as bottom electrodes [26]. Figure 3c shows the *P*–*E* hysteresis loops of the BFO film under various applied voltages. The *P*–*E* hysteresis loops were measured at a frequency of 1 kHz at room temperature. The hysteresis loops are square-like and saturated, with a polarization of 61.2 μC/cm^2^ and a coercive field of 171.6 kV/cm, revealing the high quality of the as-prepared BFO film.

**Figure 4:**
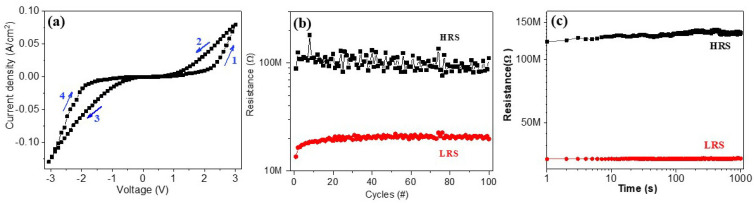
(**a**) *I*–*V* curve of the BFO/LSMO heterostructure under applied voltages from −3 V to +3 V, and (**b**) fatigue and (**c**) retention characteristics of the BFO/LSMO heterostructure. The numbers 1–4 in (**a**) indicate the sweeping sequence for the *I*–*V* curve.

A typical current–voltage (*I*–*V*) curve for the Au/BFO/LSMO heterostructure is shown in Figure 4a. *I*–*V* curves were measured by four continuous voltage sweeping sequence cycles of 0 → +3 → 0 → –3 → 0 V without an initial electrical forming process. The sweep rate was set as 1 Hz and these data were recorded on a Keithley 2450 source meter (Tektronix Inc., USA), which has a much higher current range than that measured by using a conductive AFM [23]. The observed I–V curves exhibited typical hysteresis features and a bipolar resistance state effect. In the positive voltage sweeping cycle (0 → +3 → 0 V), the heterostructure changed from a high-resistance (HR) state to a low-resistance (LR) state, whereas it changed from an LR state to a HR state during the negative voltage sweeping cycle (0 → –3 → 0 V). The heterostructure exhibited superior reliability and stability. The fatigue and retention properties were measured. The fatigue measurement was operated by the 1 ms-width ±4 V voltage pulses while the resistance was read out at 2 V. The results are shown in Figure 4b,c. The retention data were recorded by applying a pulse of 2 V, 100 ns. The resistances of the HR and LR states remained unchanged, with a steady RH/RL ratio of 10 after continuously writing/reading over 100 cycles, revealing superior fatigue characteristics, as shown in Figure 4b. Furthermore, both HR and LR keep unchanged with time, and the HR/LR ratio of the device remained approximately 8 for 10^3^ s.

To further understand the physical resistive switching mechanism, the conductive transport behaviors of the Au/BFO/LSMO heterostructure were determined. Possible conducting models, such as space-charge-limited conduction (SCLC), Poole–Frenkel emission (PF), and Schottky emission (SE), were used to fit the *I*–*V* data [27].

**Figure 5:**
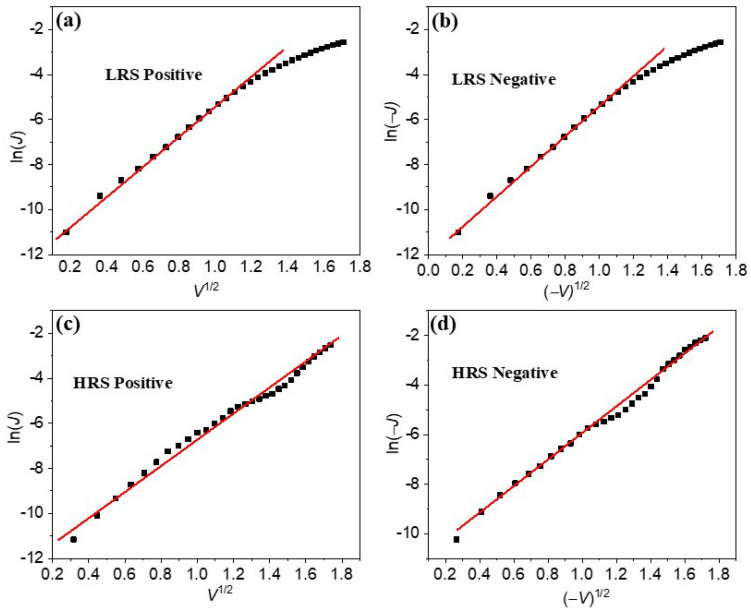
lnJ vs. *V*^1/2^ or (−*V*)^1/2^ plots of the BFO/LSMO heterostructure for LRS and HRS under positive and negative voltages. The red lines are the fitting lines for the experimental data (black scatters).

The best fit resulted from use of the Schottky emission model [28,29], which is described by

(1) $$J=AT^{2}\exp-\left[\frac{\Phi }{k_{B}T}-\frac{1}{k_{B}T}{\left(\frac{q^{3}V}{4\pi \varepsilon_{0}\varepsilon_{r}d}\right)}^{1/2}\right]$$

where *A* is the Richardson constant, Φ is the Schottky barrier height, *ε_r_* is the dielectric constant of the film, and *d* is the sample thickness. As shown in Figure 5, linear relationships between ln*J* and *V*^1/2^ indicate that the Schottky barrier controls the conductive behavior of the BFO film. In the HR state branch, linear fitting is suitable within the applied voltage range, while in the LR state branch, a linear relationship was observed in the low voltage range of 0–1.36 V.

**Figure 6:**
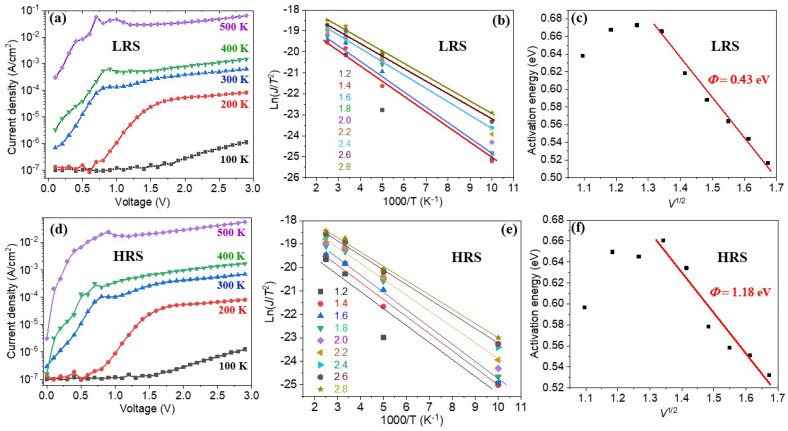
*I*–*V* curves of the BFO/LSMO heterostructure under various temperatures for LRS (**a**) and HRS (**d**), ln(*J*/*T*^2^) vs. 1000/*T* for the BFO/LSMO heterostructure measured at various voltages (**b**,**e**), and activation energy as a function of the square root of the voltage for the same film (**c**,**f**).

Figure 6a,d show the temperature dependence of the *I*–*V* curves in the range of 100–500 K. To further calculate the activation energy ($\Phi -\frac{q^{3}V}{4\pi \varepsilon_{0}\varepsilon_{r}d}$) and Schottky barrier Φ, the logarithmic of current density (ln(J/T^2^)) was plotted against the reversal temperature (1000/T) at various applied voltages, which are shown in Figure 6b,e. The activation energy was calculated to be 0.53–0.6 eV for the HR state, while it was 0.52–0.67 eV for the LR state. These activation energy values are identical to the activation energy of oxygen vacancies in ferroelectric oxides [30]. Furthermore, the activation energies were replotted against the square root of the voltage, as shown in Figure 6c,f. The effective barrier heights between the film and electrode were calculated as 0.43 eV and 1.18 eV for the LR and HR states, respectively. One may note that there is a slight deviation of the linear fitting in the low temperature range. In the low voltage range, the applied voltage is lower than the coercive field of the BFO film, which cannot reverse the polarization and thus results in a different barrier height. Moreover, the point defects such as bismuth and/or oxygen vacancies coupled with ferroelectric polarization would also affect the effective barrier height [31]. For a fuller understanding of it, more complex modeling may be required to accurately describe the nonlinear behavior in the entire voltage range.

According to the above results, the resistive switching properties in the BFO/LSMO heterostructure can be interpreted by considering the modulation effect on the depletion width and the potential barrier height via ferroelectric polarization reversal, along with the oxygen vacancies migration near the BFO/LSMO interface [32,33]. The work functions of LSMO and Au are 4.7 eV and 5.1 eV, respectively [9,34], while the work function, electron affinity, and energy band gap of BFO are 4.7 eV, 3.3 eV, and 2.8 eV, respectively [9,35]. Thus, the nominal Schottky barrier heights for the Au/BFO and the LSMO/BFO contacts can be estimated as 1.8 eV and 1.5 eV, respectively. However, one may also note that ferroelectric polarization also plays a key role in controlling the transport properties of metal/ferroelectric/metal structures.

**Figure 7:**
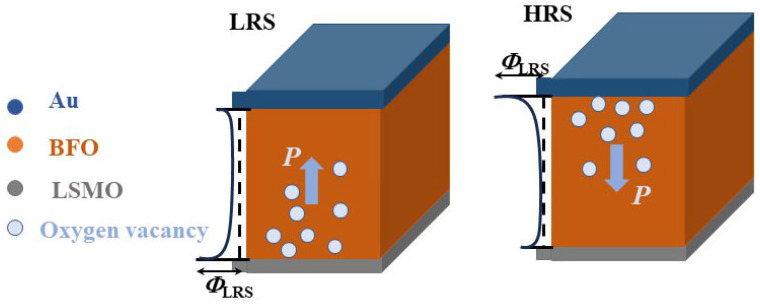
Schematic diagrams of the Au/BFO/LSMO heterostructure in up and down polarization states.

The conduction mechanism was discussed by only considering the barrier-height modulations by ferroelectric polarization [23]. However, the migration of the oxygen vacancies should be taken into consideration for further understanding the conduction mechanism. As shown in the schematic diagrams in Figure 7, with upward polarization, the downward depolarization field induces electron accumulation near the BFO/LSMO interface, reducing the width depletion region on the BFO side and thus the barrier height. Additionally, with upward polarization in the BFO layer, the Schottky barrier at the BFO/LSMO interface is the dominating factor in controlling the transport property in the Au/BFO/LSMO heterostructure. When applying a positive bias voltage, the polarization was switched downwards and the carriers were trapped near the Au/BFO interface, which then enhanced the barrier near the top Au/BFO interface. Therefore, the resistance of the heterostructure with upward polarization (downward polarization) is in an LR (HR) state. Furthermore, oxygen vacancies are frequently observed in BFO thin films [36,37] and the migration and distribution of oxygen vacancies also lead to band bending and further increase the Schottky barrier height. As shown in the schematic in Figure 7, oxygen vacancies drift toward and accumulate at the BFO/LSMO and Au/BFO interface for upwards and downwards polarization, respectively, which further enhances the Schottky barrier height for each interface, leading to LR and HR states, respectively. The proposed scenario was confirmed by the above I–V curve fitting, as well as the calculated Schottky barrier values of 0.43 eV and 1.18 eV for the LRS and HRS in Figure 6, respectively.

## 4. Conclusions

In summary, we have reported ferroelectric-related resistive switching phenomena in an Au/BFO/LSMO heterostructure. Stable bipolar resistive switching behavior was confirmed by *I*–*V* hysteresis loops. The conduction mechanism was revealed to follow the Schottky emission model, and effective Schottky barriers of 0.43 eV and 1.18 eV under HR and LR states were estimated based on temperature-dependent current–voltage curves. The observed resistive switching characteristics were determined by the modulation effect on the depletion region width and the potential barrier height via ferroelectric polarization reversal, along with the oxygen vacancies migration at the BFO/LSMO and Au/BFO interfaces.

## Data Availability

The data presented in this study are available on request from the corresponding author.
